# Intelligent Fault Diagnosis and Forecast of Time-Varying Bearing Based on Deep Learning VMD-DenseNet

**DOI:** 10.3390/s21227467

**Published:** 2021-11-10

**Authors:** Shih-Lin Lin

**Affiliations:** Graduate Institute of Vehicle Engineering, National Changhua University of Education, No.1 Jin-De Road, Changhua City 50007, Taiwan; lin040@cc.ncue.edu.tw

**Keywords:** VMD-DenseNet, intelligent fault diagnosis, bearing fault

## Abstract

Rolling bearings are important in rotating machinery and equipment. This research proposes variational mode decomposition (VMD)-DenseNet to diagnose faults in bearings. The research feature involves analyzing the Hilbert spectrum through VMD whereby the vibration signal is converted into an image. Healthy and various faults show different characteristics on the image, thus there is no need to select features. Coupled with the lightweight network, DenseNet, for image classification and prediction. DenseNet is used to build a model of motor fault diagnosis; its structure is simple, and the calculation speed is fast. The method of using DenseNet for image feature learning can perform feature extraction on each image block of the image, providing full play to the advantages of deep learning to obtain accurate results. This research method is verified by the data of the time-varying bearing experimental device at the University of Ottawa. Through the four links of signal acquisition, feature extraction, fault identification, and prediction, a mechanical intelligent fault diagnosis system has established the state of bearing. The experimental results show that the method can accurately identify four common motor faults, with a VMD-DenseNet prediction accuracy rate of 92%. It provides a more effective method for bearing fault diagnosis and has a wide range of application prospects in fault diagnosis engineering. In the future, online and timely diagnosis can be achieved for intelligent fault diagnosis.

## 1. Introduction

With the development of modern machinery and equipment, the structure of equipment has become more complex. The failure of parts in machinery and equipment may cause the entire equipment to fail to operate, and the failure of key parts may cause serious casualties and economic losses. The mechanical fault diagnosis technology has matured, and its results have been widely used in industrial production. However, with the emergence and widespread application of advanced technologies such as sensors, big data, and the Internet of Things, the development trend of mechanical fault diagnosis technology is bound to be combined with contemporary cutting-edge technologies. These factors promote the transformation of the monitoring and diagnosis of industrial equipment faults to the direction of intelligence; the future development direction of this technology combines with artificial intelligence.

Rolling bearing fault diagnosis is the process of determining the damage state through detection, isolation, and identification through data collected by the health monitoring of the rolling bearing. The early fault diagnosis method of rolling bearing was relatively simple, mainly through some statistical parameters (average value, root mean square value, kurtosis, etc.) to judge the fault condition of rolling bearing. However, these statistical values cannot determine the noise and interference caused by shaft speed changes, gears, and other vibration sources. Cempel [1] constructed a set of discriminants for the crest factor, pulse factor, harmonic factor, frequency modulation factor, and other parameters of the random vibration process. Sturm et al. [2] designed a zero-mean normalization parameter and found that the parameter normalized to zero-mean condition is more suitable for fault diagnosis than the absolute value of the time-domain parameter. Martin et al. [3] used standardized skewness and standardized kurtosis to determine early failures of rolling bearings. Pand et al.’s [4] research extended the field even further and proposed the use of statistical moments to detect the health status of rolling bearings. Following this, the industry explored parameterized signal processing methods. For example, Mechefske et al. [5] found that the effect of parameterized spectral index fault classification is better than that of traditional fast Fourier transform. Logan and Mathew [6] have proposed a correlation integration algorithm to measure the results of the correlation di-mension on the rolling bearing test rig. Vapnik et al. [7] proposed a learning theory based on statistics. It showed superiority in the identification of small samples and non-linear processing, which was later introduced into the field of fault diagnosis and achieved wide applications. Li et al. [8] proposed a bearing vibration feature extraction method based on multi-scale permutation entropy and binary tree based on an improved support vector machine. Lin [9] analyzed the impact of different Gaussian kernel functions, such as fine, medium, and coarse, on the performance of the SVM algorithm in the classification results of related motor data sets obtained by motor fault detection and diagnosis. The most critical part of bearing fault diagnosis is to effectively process the collected vibration signals to obtain features that can express bearing state information, that is, feature extraction, which lays a good foundation for subsequent fault pattern recognition. Vibration signal processing methods generally include three types of analysis: time domain analysis, frequency domain analysis, and time-frequency domain analysis [10]. Empirical mode decomposition can adaptively decompose non-stationary signals based on the time scale. It is a signal processing method that is widely used in the field of mechanical fault diagnosis [11,12]. Han et al. [13] proposed a new power-based IMF selection algorithm and used an improved fully integrated EMD with adaptive noise and a multilayer perceptron neural network to verify the performance of the proposed fault diagnosis system. Lee and Hung [14] proposed a feature ranking and differential evolution method for feature selection in brushless DC motor BLDC fault diagnosis. This research uses Hilbert–Huang transform (HHT) to extract the hall signal characteristics of four different types of brushless DC motors. Wang [15] proposed to use short-time Fourier transform (STFT) to preprocess the original signal to obtain the corresponding time-frequency diagram. Then, a convolutional neural network (CNN) began to be used to adaptively extract the features of the time-frequency image. Li [16] proposed pseudo-Wigner–Ville distribution and relative cross information methods for intelligent fault diagnosis methods for motor roller bearings running at unsteady speed and load. Gu et al. [17] developed a new type of long short-term memory (LSTM) model with discrete wavelet transform (DWT) for multi-sensor fault diagnosis. Nguyen’s [18] research applies the wavelet vibration imaging method (WVI) to the denoised vibration signal. The obtained scale map is used as the input of the deep convolutional neural network architecture (DCNA), which is used to extract the discriminative features in the gearbox and the multiple degree tooth failure (MDTF) classification under variable speed conditions. Dragomiretskiy et al. [19] proposed variational mode decomposition, which is a new non-recursive, variational adaptive signal processing method. The decomposition process is the process of solving the variational problem. Specifically, it first constructs the variational problem for the input signal. Then, by solving the variational problem, the signal is decomposed into a specified number of eigenmode functions. In addition, variational mode decomposition has gained attention and application in bearing fault diagnosis [20]. Lin’s research proposed an automatic fault diagnosis system combining VMD and ResNet101 for bearing fault diagnosis. Recent efforts have also been made in the field of deep learning to promote the miniaturization of neural networks. While ensuring the accuracy of the model, it is smaller and faster and will become a popular method in the future. These models make it possible for mobile terminals and embedded devices to run neural network models. Online and timely diagnosis can be achieved in intelligent fault diagnosis. This research proposes a comparison of advanced lightweight network models such as ShuffleNet, MobileNet, and DenseNet as traditional deep learning networks are large, slow, and complicated. Lin’s research, for example, uses the Federal University of Rio de Janeiro database, a traditional fixed speed database. The database of this research is special the speed of each data is different from the traditional fixed speed, yet the database is more challenging. Traditional machine learning methods such as artificial neural networks, sparse representation, fuzzy inference, SVM have been widely applied in bearing fault diagnosis [21,22,23]. Recent years have seen the advancement of training deep network technology and a substantial increase in hardware computing capabilities. Deep learning and machine learning technologies have more powerful feature extraction and processing capabilities, as well as wide applicability and model migration capabilities; this superior performance makes them widely used in various industries. Methods based on deep learning have gradually become the focus of attention, and related fault diagnosis research is shown in the literature [24,25,26,27,28]. In a paper published by Google, the MobileNet lightweight network was proposed. The MobileNet deep convolutional neural network is mainly developed for mobile terminals or embedded devices [29]. Compared with traditional convolution, MobileNet uses depth separable convolution to divide the convolution operation into two parts, Depthwise and Pointwise. The calculation amount of depth separable convolution can be eight to nine times less than that of traditional convolution. The design goal of ShuffleNet also includes how to use limited computing resources to achieve the best model accuracy, which requires a good balance between speed and accuracy [30]. The core of ShuffleNet uses two operations: pointwise group convolution and channel shuffle, which greatly reduces the amount of model calculations while maintaining accuracy. After ResNet, Huang [31] proposed the DenseNet network, which inherited the idea of residual network and improved the connection method. The DenseNet network takes image features as the starting point, and achieves better results, and reduces a large number of parameters through the reuse of image features.

Traditional machine learning or deep learning classification prediction requires the selection of features. The features to be used and the number of features are determined and selected according to the intended use; there is no fixed standard operating procedure. The spectrogram analyzed by VMD can fully present the characteristics of bearing diagnostic signals. Through image classification and prediction in deep learning, ResNet is a good method of classification and prediction. Compared with convolutional neural networks and general deep learning methods, ResNet can solve the problems, to an extent, of gradient descent and gradient disappearance. It produces it as the number of layers increases, and each layer has a corresponding weight, and the number of parameters will increase accordingly. In order to achieve real-time bearing monitoring and diagnosis, it is necessary to reduce the amount of network calculations. In recent years, DenseNet has been proposed. DenseNet is also an improved neural network framework based on convolutional neural networks, which is mainly composed of dense blocks, transition layers, and bottleneck layers. In order to further improve the efficiency of information flow between the various layers, DenseNet proposes a different connection method, that is, the direct connection from which layer to all subsequent layers is introduced. It enhances the propagation of features to promote the repetition and effective use of features, reduces the number of parameters, and simultaneously reduces the calculation. Therefore, VMD spectrogram plus DenseNet is suitable for bearing fault diagnosis.

The research contribution aims to use VMD to analyze the Hilbert spectrum, convert the one-dimensional bearing signal into a two-dimensional time-frequency graph, and combine it with the deep neural network DenseNet to realize intelligent fault classification prediction and diagnosis. Generally, neural networks require high-intensity calculations, but for small and medium-sized embedded systems, computing resources are limited. In order to deploy the network model in a small embedded system, it mainly compresses the large-scale classical classification network model and reduces the number of parameters of the model operation so that it can run in the case of insufficient CPU, memory, or other hardware resources.

## 2. Research Methodology

### 2.1. VMD

VMD is a new signal processing method, which is different from other separation methods and is mainly reflected in the process of solving the center frequency and bandwidth of each component. The basic principle of VMD is to use Wiener filtering and Hilbert transform to construct multiple constraint problems from an input signal. By continuously updating the bandwidth and center frequency of each constraint problem to solve the problem, the adaptive decomposition of the vibration signal is finally realized.

The bearing vibration signal is split into *K* IMF components by the constrained variational model. Its intermediate frequency range and bandwidth are quickly updated in the iterative loop process so that the sum of the frequency domain widths of the *K* IMF components finally obtained is the smallest. At the same time, the addition of *K* IMF components can restore the original vibration signal. The summary of VMD theory is as follows [19]. Each IMF component can be functionalized into an amplitude modulation-frequency modulation mode function $u_{k}\left(t\right)$, as shown in the following formula:

(1) $$u_{k}\left(t\right)=A_{k}\left(t\right)\cos\left(\phi_{k}\left(t\right)\right)$$

Here, $A_{k}\left(t\right)$ is taken as the instantaneous amplitude of $u_{k}\left(t\right)$, and $A_{k}\left(t\right)$ ≥ 0; (*t*) instantaneous frequency. $\phi_{k}\left(t\right)$ is used as the instant phase of $u_{k}\left(t\right)$, and $\phi_{k}\left(t\right)$ is the first-order differential of *t* to obtain the instantaneous frequency of $u_{k}\left(t\right)$:

(2) $$w_{k}\left(t\right)=\frac{\phi_{k}\left(t\right)}{dt},w_{k}\left(t\right)\geq 0$$

The goal of estimating the frequency domain width of each IMF component by creating a variational pattern:

(1) Conduct Hilbert transformation on the mode function $u_{k}\left(t\right)$. Acquire its analysis input:

(3) $$\left(\delta \left(t\right)+\frac{j}{\pi t}\right)*u_{k}\left(t\right)$$

(2) Use the transformation parameter $e^{-j\omega_{k}t}$ to adjust the frequency domain of each mode mapping to their initial band:

(4) $$\left[\left(\delta \left(t\right)+\frac{j}{\pi t}\right)*u_{k}\left(t\right)\right]e^{-j\omega_{k}t}$$

(3) Derive the norm gradient square $L^{2}$ in Equation (4), estimate the width of $u_{k}\left(t\right)$ mode function, and the initial variational constraint problem:

(5) $$\{\begin{array}{c} \underset{\left\{u_{k}\right\},\left\{\omega_{k}\right\}}{min}\left\{\overset{K}{\underset{k=1}{\sum }}\left|\right|\partial_{t}\left[\left(\delta \left(t\right)+\frac{j}{\pi t}\right)*u_{k}\left(t\right)\right]e^{-j\omega_{k}t}{\left|\right|}_{2}^{2}\right\}\\ s.t.\;\overset{K}{\underset{k=1}{\sum }}u_{k}\left(t\right)=f\left(t\right) \end{array}\;$$

where $\left\{u_{k}\right\}$ stands for IMF_1_-IMF_K_, $\left\{u_{k}\right\}=\left\{u_{1},u_{2},\ldots u_{k}\right\}$. $\left\{\omega_{k}\right\}$ represents the bilateral symmetric frequency of IMF_1_-IMF_K_, $\left\{u_{k}\right\}=\left\{u_{1},u_{2},\ldots u_{k}\right\}$. δ(t) is the average pulse function; $\partial_{t}$ is the first-order partial derivative of the functional with respect to time *t*; *j* is the imaginary unit; ∗ is the convolution symbol. The amplified Lagrangian functional *L* is introduced and the restricted variational target is converted in Equation (5) into an unrestricted variational target for analysis, as shown in the formula:

(6) $$L\left(\left\{u_{k}\right\},\left\{\omega_{k}\right\},\lambda \right)=\alpha \overset{K}{\underset{k=1}{\sum }}\left|\right|\partial_{t}\left[\left(\delta \left(t\right)+\frac{j}{\pi t}\right)*u_{k}\left(t\right)\right]e^{-j\omega_{k}t}{\left|\right|}_{2}^{2}+\left|\right|f\left(t\right)-\overset{K}{\underset{k=1}{\sum }}u_{k}{(t)\left|\right|}_{2}^{2}+\langle \lambda \left(t\right),f\left(t\right)-\overset{K}{\underset{k=1}{\sum }}u_{k}\left(t\right)\rangle$$

In the formula, *α* is the secondary punishment factor. When white noise exists, its existence can ensure the reconstruction accuracy of the original signal. In the formula, *α* is the secondary punishment factor. *λ*(*t*)—Lagrange factor multiplier, the control limits used to determine the factor are all executed in place. The actualized circulation regression multiplier algorithm derives the expanded Lagrange map of Equation (6). The detailed export process is as follows:

(1) Initial setup $\left\{{\hat{u}}_{K}^{1}\right\}$, $\left\{\omega_{K}^{1}\right\}$, ${\hat{\lambda }}^{1}$ *n*;

(2) Implement outer loop *n* = *n* + 1;

(3) If *k* = 1:*K*, implement this inner loop;

If there are *ω* ≥ 0, reiterate the functional ${\hat{u}}_{k}$ for each of them

(7) $${\hat{u}}_{k}^{n+1}\left(\omega \right)=\frac{\hat{f}\left(\omega \right)-\sum_{i\neq k}\hat{u}\left(\omega \right)+\frac{\hat{\lambda }\left(\omega \right)}{2}}{1+2\alpha {\left(\omega -\omega_{k}\right)}^{2}}$$

Iterate the functional again *ω_k_*:

(8) $$\omega_{k}^{n+1}=\frac{\int_{0}^{\infty }\omega {\left|{\hat{u}}_{k}\left(\omega \right)\right|}^{2}\;d\omega }{\int_{0}^{\infty }{\left|{\hat{u}}_{k}\left(\omega \right)\right|}^{2}\;d\omega },$$

(4) Update *λ*:

(9) $${\hat{\lambda }}^{n+1}\left(\omega \right)\leftarrow {\hat{\lambda }}^{n}\left(\omega \right)+\tau \left(\hat{f}\left(\omega \right)-\underset{k\;}{\sum }{\hat{u}}_{k}^{n+1}\left(\omega \right)\right),$$

In the formula, *τ* is the noise tolerance scale. If the signal source has large background noise, then setting *τ* = 0 can achieve outstanding noise reduction purposes.

(5) Continuously run the process (2)–(4), when the following conditions can be reached:

(10) $$\overset{K}{\underset{k}{\sum }}\left|\right|{\hat{u}}_{k}^{n+1}-{\hat{u}}_{k}^{n}{\left|\right|}_{2}^{2}/\left|\right|{\hat{u}}_{k}^{n}{\left|\right|}_{2}^{2}<\epsilon$$

When the loop is paused, *K* IMF components with the smallest total bandwidth are obtained.

### 2.2. DenseNet

With the deepening of the convolutional neural network structure, new problems have appeared. After multi-layer transmission, the input information and gradient information may have been lost or disappeared when they reach the end of the network. In order to solve the degradation problem of deep convolutional neural networks, He Kaiming et al. [32] proposed ResNet. Traditional convolutional neural networks use parameterized layers to directly map between input and output, while the residual structure used by ResNet uses multiple parameterized layers to learn the residuals between input and output. By learning the residuals, the network converges faster, and because more layers of parameters are used, the accuracy of the network is also improved.

Suppose $X_{n}$ is the output of the nth layer of the convolutional neural network, and $H_{n}$ is the non-linear transformation composite function of the *n*-th layer. The composite function is a combined operation, including batch normalization (BN), rectified linear unit (ReLU), and convolution or pooling. The combined operation of convolution or pooling, the output $X_{n-1}$ of the *n* − 1 th layer in the traditional convolutional neural network is the input of the *n*-th layer:

(11) $$X_{n}=X_{n-1}$$

Because of the residual structure of ResNet, the output of the *n*-th layer is affected by the input of the previous layer, which can be expressed as:

(12) $$X_{n}=H_{n}(X_{n-1})+X_{n-1}$$

In ResNet, the output of the *n*-th layer is connected by summation, which may affect the spread of information in the network.

For the problem of vanishing gradient, many researchers have provided solutions, in addition to ResNet, there are network structures such as Highway Networks [33], Stochastic Depth [34], and FractalNets [35]. Although these network structures are different, they are all based on the idea of mapping low-level feature maps to high-level networks. Along this line, Huang et al. proposed a densely connected convolutional neural network, DenseNet [31]. Compared with ResNet, DenseNet is a bolder and densely connected network created to obtain better anti-fitting characteristics. DenseNet connects all layers to each other, each layer receives all the previous layers as its new input to ensure that the most inter-layer information is transmitted. DenseNet’s connection method is called a dense block. Compared with other networks, the number of output feature maps of each convolutional layer in the dense block is small, which also makes DenseNet’s network narrower with fewer parameters. This densely connected method makes the transmission of feature maps and gradients more efficient, so the network will be easier to train. Compared with other deep networks that have the problem of gradient disappearance caused by the transmission of input information and gradient information in many layers, DenseNet’s connection method allows each layer to directly connect the input information and loss function, which can effectively reduce the problem of gradient disappearance. In DenseNet, because the back layer will connect all the front layers as input, for an *n*-layer network, there are a total of *n*(*n* + 1)/2 connections, and the output of the nth layer is:

(13) $$X_{n}=H_{n}([X_{0},X_{1},\cdots X_{n-1}]),$$

where [$X_{0},X_{1},\cdots X_{n-1}]$ represents the stitching of the 0-th, …, *n* − 1 th layer output feature maps. The following summary illustrates the DenseNet methodology [31] in this study. The network structure of DenseNet is mainly composed of DenseBlock and Transition.

Composite function: Here $H_{n}\left(\cdot \right)$ is defined as a combined function of three consecutive operations: BN, followed by a ReLU and a 3 × 3 convolution (Conv).

Pooling layers: When the size of the feature map changes, there will be problems with the wiring operation in Equation (13). However, convolutional networks have a basic partial down-sampling layer, which can change the size of the feature map. In order to facilitate the implementation of down-sampling, the network is divided into multiple densely connected dense blocks. The layer between each block is called the transition layer, which completes the convolution kernel pooling operation.

Growth rate: If each function $H_{n}\;$ generates *k* feature maps, the subsequent l layer will have $k_{0}+k\times \left(n+1\right)$ feature maps as input, where $k_{0}$ represents the number of channels in this layer. An important difference between DenseNet and the existing network structure is that the network of DenseNet is narrow, such as *k* = 12. The super argument *k* is called the growth rate of the network.

Bottleneck layer: Although each layer only produces k output feature maps, it has more inputs. Adding 1 ∗ 1 convolution before the 3 ∗ 3 convolution in the bottleneck layer to achieve dimensionality reduction can reduce the amount of calculation. This design is effective for DenseNet, that is, the structure of BN-ReLU-Conv(1 ∗ 1)-BN-ReLU-Conv(3 ∗ 3) is called DenseNet-B.

Compression: In order to simplify the model, the number of feature maps is reduced in the transition layer. If a dense block has m feature maps, this will allow the subsequent transition layer to generate $\theta_{m}$ output feature maps. Among them, 0<θ≤1 represents the Compression coefficient. When *θ* = 1, the number of feature maps passing through the transition layer does not change.

**Figure 1:**
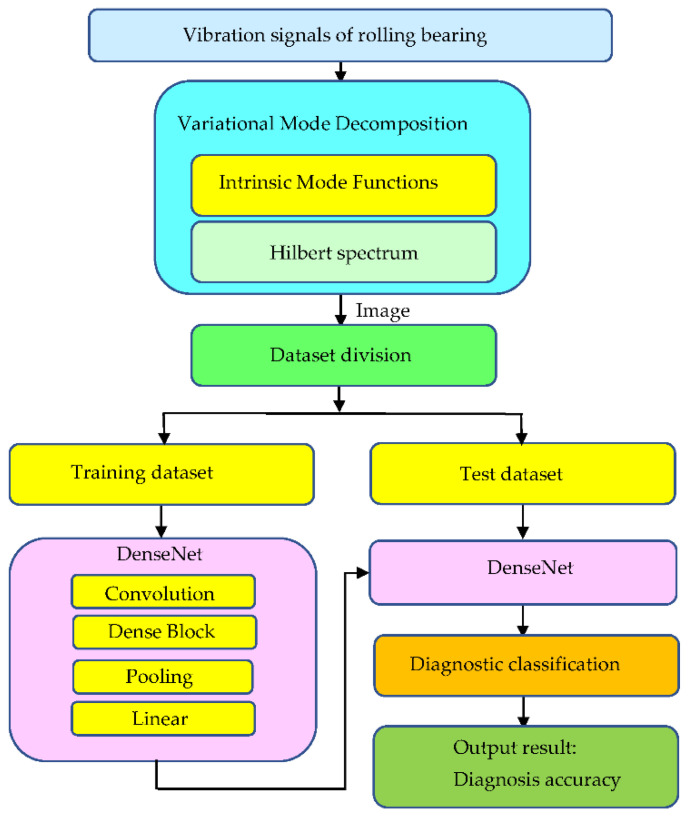
The entire flow chart of VMD-DenseNet.

Here we explain the entire flow chart of VMD-DenseNet and how to implement it. Figure 1 shows the entire flow chart of VMD-DenseNet. Vibration signals of rolling bearing health and failure are obtained from the motor test platform. VMD is analyzed into IMF through algorithm and converted into Hilbert spectrum image. The images are divided into a training database and a test verification database. The training database is entered into DenseNet for training classification, using convolution, dense block, pooling, and linear as described in the previous paragraph. The training result model is provided to the test database for test verification. DenseNet performs diagnostic classification based on test data and provides the accuracy of diagnostic classification.

## 3. Database Description

**Figure 2:**
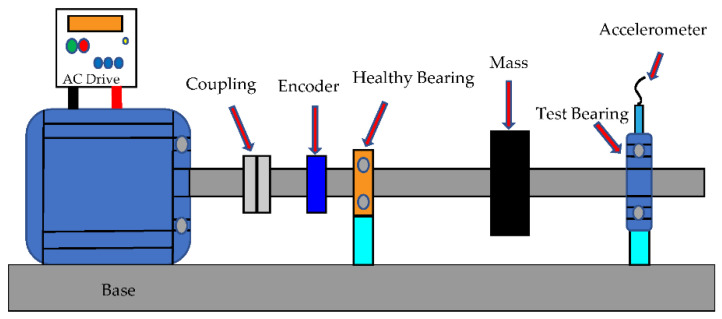
The Experimental platform of the University of Ottawa bearing test rig for a ball bearing system.

The data obtained in this study provide test data for healthy and faulty motors, obtained from the University of Ottawa website at https://data.mendeley.com/datasets/v43hmbwxpm/2 (accessed on 9 November 2021). The data is collected from the vibration signals of bearings with different health conditions under time-varying speed conditions. The experimental setup is shown in Figure 2. For each data set, there are two experimental setting conditions in the bearing health status and the changing speed status. The health and failure conditions of the bearing include (1) health, (2) inner ring defects, (3) outer ring defects, (4) ball defects, and (5) composite defects including inner ring, outer ring, and a ball. The operating speed conditions are (i) increase speed, (ii) decrease speed, (iii) increase and then decrease speed, and (iv) decrease and increase speed. Therefore, there are 20 different situations. In order to ensure the repeatability of the data, three trials were collected for each experimental setting, resulting in a total of 60 data sets. Table 1 shows the bearing health or failure and test conditions. Each data set contains the vibration data measured by the two-channel accelerometer and the rotational speed data measured by the encoder. The data are acquired by the NI data acquisition boards (NIUSB-6212BNC); the bearing type is ER16K. All data are sampled at 200,000 Hz, and the sampling duration is 10 s. The CPR (cycles per revolution) of the encoder is 1024.

## 4. Results and Discussion

The process of VMD involves three very important theories: Wiener filtering, Hilbert transform, and frequency mixing. The basic principle of VMD uses Wiener filtering and Hilbert transform to construct multiple constraint problems from an input signal and to solve the constraint problem by continuously updating the bandwidth and center frequency of each constraint problem. Finally, the adaptive decomposition of the vibration signal is realized because the VMD method uses a non-recursive, variational adaptive decomposition mode. Therefore, it can effectively solve the problems of mode aliasing and end effect in other commonly used mechanical fault vibration signal processing methods. In addition, the VMD method has the advantages of fast running speed and stable decomposition results.

The VMD parameter setting uses Max Iterations, one of the optimizations’ stopping criteria; the optimization of Max Iterations is stopped when the number of iterations is greater than 600, the maximum number of optimization iterations of 600. Num IMF (the number of extracted IMFs) is 5 IMF, Initial IMFs (initial IMF) is a zero matrix, and Penalty Factor (penalty factor) is 1500. This parameter determines the fidelity of reconstruction. Using a smaller penalty factor value can obtain tighter data fidelity. LMU update Rate (the update rate of the Lagrangian multiplier) is 0.01, which is the update rate of the Lagrangian multiplier in each iteration. A higher rate will lead to faster convergence, but it will increase the optimization process into a local, best opportunity. The initialize method is peaked, and peaks initialize the center frequency to the peak position of the signal in the frequency domain.

**Figure 3:**
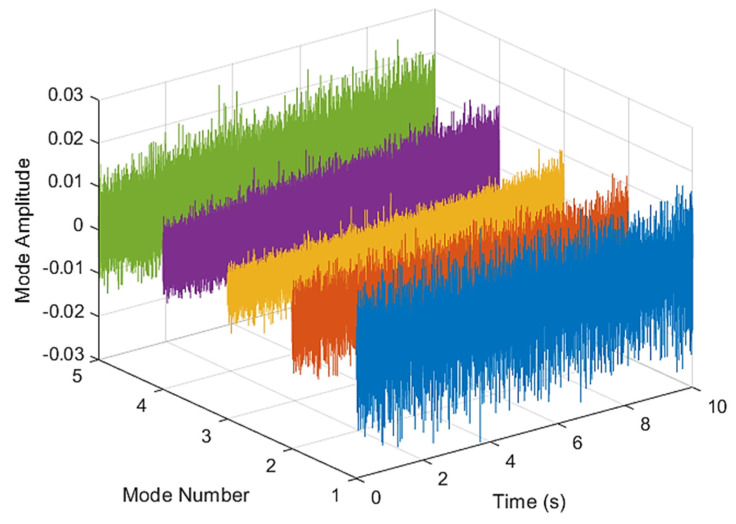
VMD analysis from a healthy bearing under increasing rotational speed condition in time domain.

**Figure 4:**
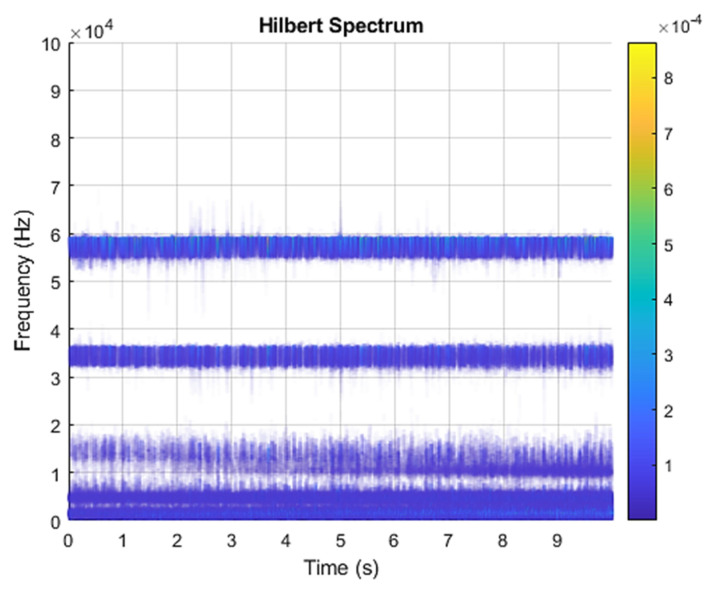
VMD analysis from a healthy bearing under increasing rotational speed condition in Hilbert spectrum.

This result discusses the application of the VMD method to the actual bearing vibration signal, and for the healthy state of the rolling bearing as well as the different positions of the inner ring, outer ring, and rolling element mixing (acceleration, deceleration, acceleration and deceleration, and deceleration and acceleration). The four speed-increasing modes are tested experimentally using the VMD method. Figure 3 and Figure 4 show the VMD analysis of the healthy bearing state. The motor speed is increased from 846 RPM to 1428 RPM. Figure 3 is the time-domain waveform diagram of the vibration signal, Figure 3 and Figure 4 show the VMD analysis of the healthy bearing state. The motor speed is increased from 846 RPM to 1428 RPM. Figure 3 is the time-domain waveform diagram of the vibration signal VMD. Figure 4 is a component spectrum diagram; each state vibration signal is decomposed into five mode components. The results show that the IMF Hilbert marginal spectrum of the vibration data processed by VMD has a higher frequency resolution. There are five frequencies in healthy bearings, the most obvious being 57 k Hz, 35 k Hz, 15 k Hz, 5 k Hz, 1.6 k Hz. The healthy bearing has not changed due to the increase in speed. The healthy bearing has four transmission modes: increase, deceleration, increase and then decelerate, and deceleration and increase again. Each mode contains data with three measurements, and the speed is measured each time. There are a total of 12 different test data; these data are all converted into images of Hilbert’s marginal spectrum.

**Figure 5:**
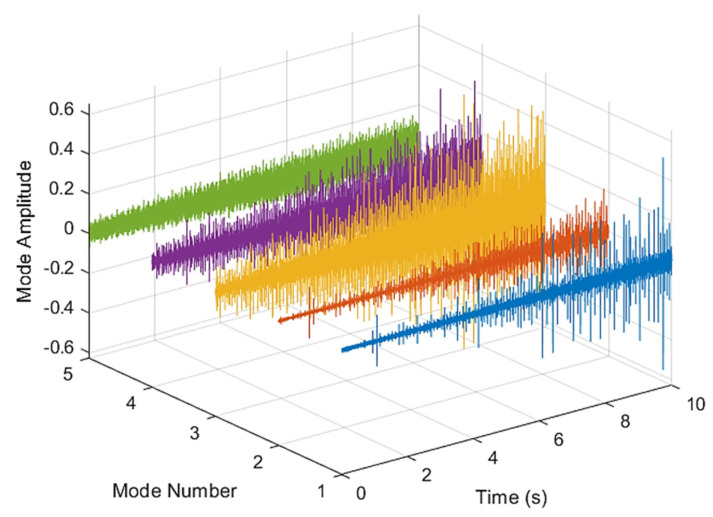
VMD analysis from an inner race fault bearing under increasing rotational speed condition in time domain.

**Figure 6:**
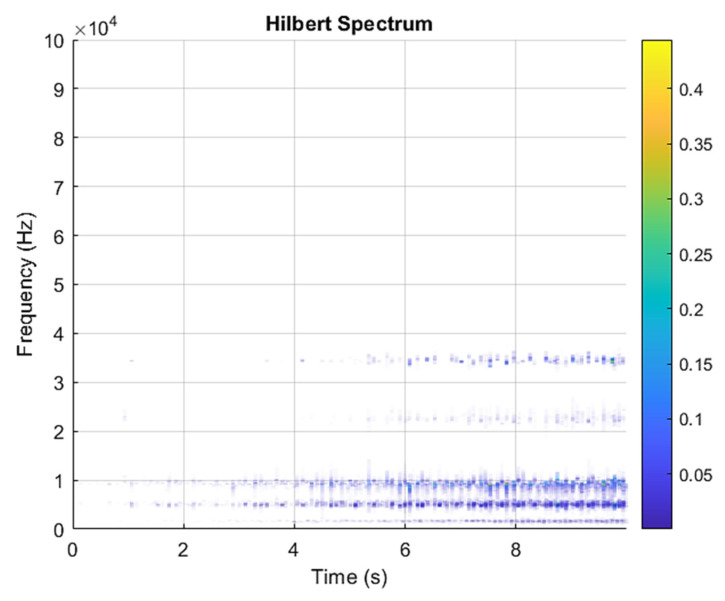
VMD analysis from an inner race fault bearing under increasing rotational speed condition in Hilbert spectrum.

Figure 5 and Figure 6 are the VMD analysis results of the inner race fault bearing state, and Figure 4 is the time-domain waveform diagram of the VMD of the vibration signal. Figure 6 is a component spectrum diagram; each state vibration signal is decomposed into five mode components. The results show that the IMF Hilbert marginal spectrum of the vibration data processed by VMD has a higher frequency resolution. There are five frequencies in healthy bearings, the most obvious are 35 k Hz, 23 k Hz, 9 k Hz, 5.4 k Hz, 1.9 k Hz. The higher the speed of the faulty bearing, the greater the vibration. The inner race fault bearing also has four transmission modes: increase, decrease, increase and decrease, and decrease and increase. Each mode has three measurements. The rotation speed is different during each measurement, and there is a total of 12 test data; these data are all converted into images of Hilbert’s marginal spectrum.

**Figure 7:**
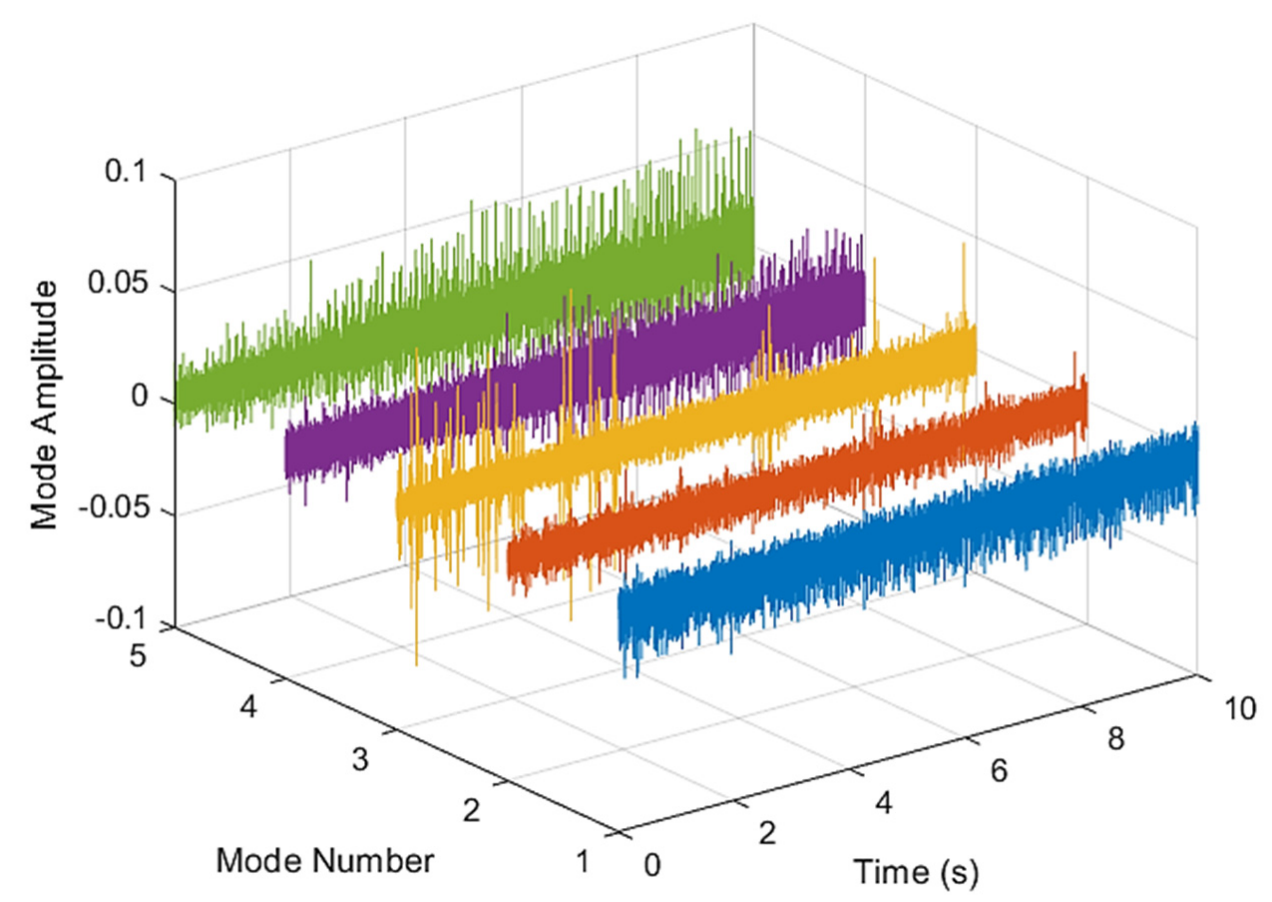
VMD analysis from an outer race fault bearing under increasing rotational speed condition in time domain.

**Figure 8:**
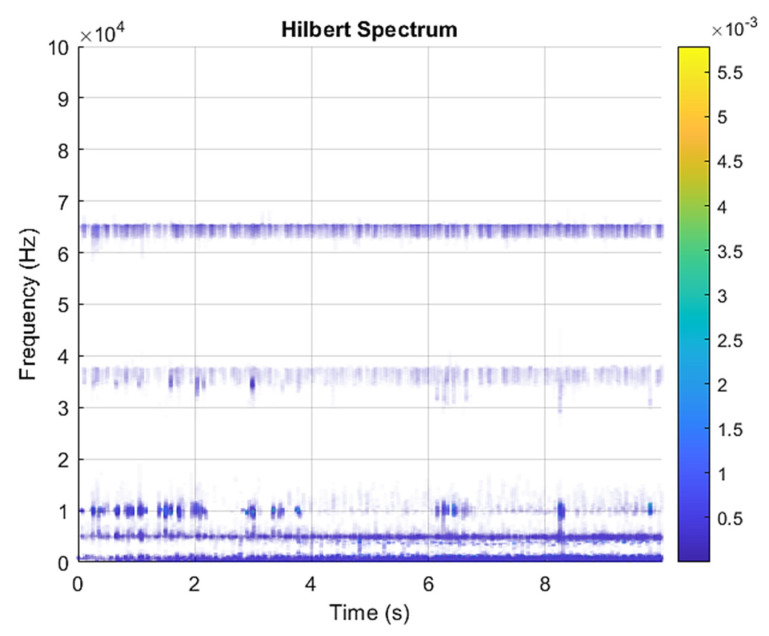
VMD analysis from an outer race fault bearing under increasing rotational speed condition in Hilbert spectrum.

Figure 7 and Figure 8 show the VMD analysis results of the outer race fault-bearing state. Among them, Figure 6 is the time-domain waveform of the vibration signal variational mode decomposition. Figure 8 is the component spectrogram. Each state vibration signal is decomposed into five mode components. The results show that the IMF Hilbert marginal spectrum of the vibration data processed by VMD has a higher frequency resolution. There are five frequencies in healthy bearings, the most obvious being 65 k Hz, 37 k Hz, 10 k Hz, 5 k Hz, 750 Hz. The higher the speed of the faulty bearing, the greater the vibration. The outer race fault bearing also has four speed modes: increase, decrease, increase and decrease, and decrease and increase. There are three measurements for each mode, and the rotation speed is different during each measurement. There are 12 test data in total; these data are all converted into images of Hilbert’s marginal spectrum.

**Figure 9:**
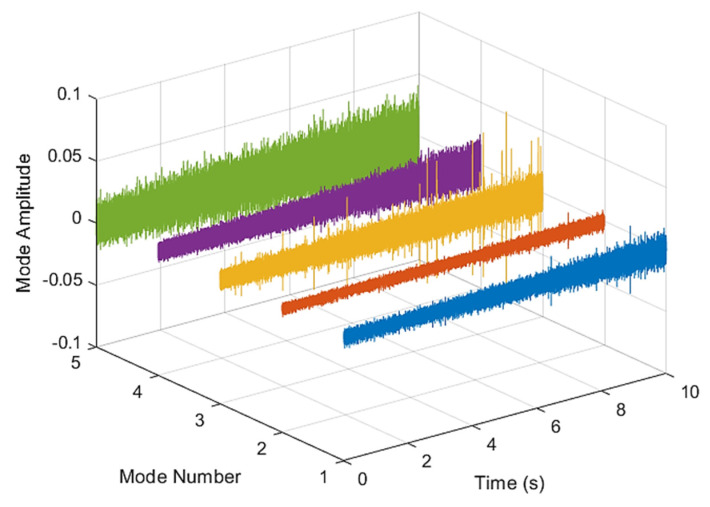
VMD analysis from a ball fault bearing under increasing rotational speed condition in time domain.

**Figure 10:**
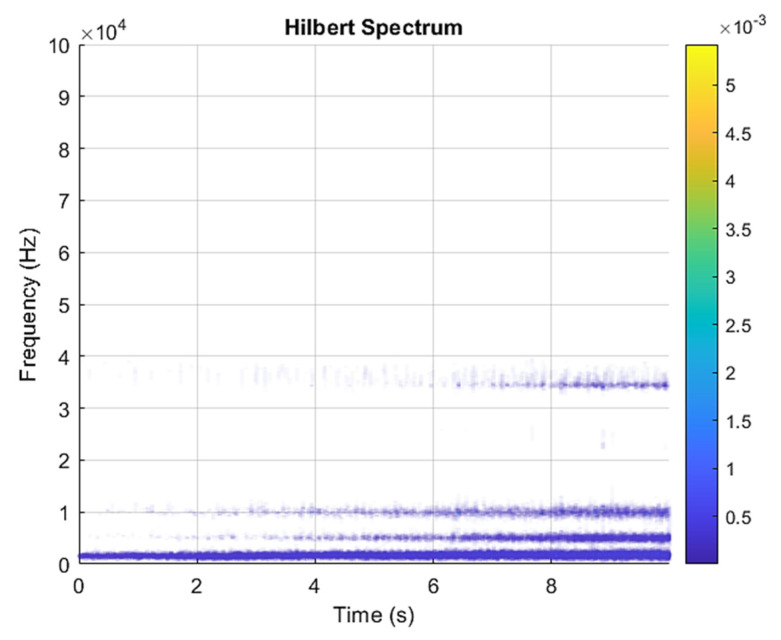
VMD analysis from a ball fault bearing under increasing rotational speed condition in Hilbert spectrum.

Figure 9 and Figure 10 are the VMD analysis results of the ball fault-bearing state. Figure 9 is the time-domain waveform of the vibration signal’s VMD. Figure 10 is the component frequency spectrum. The results show that the IMF Hilbert marginal spectrum of the vibration data processed by VMD has a higher frequency resolution. There are five frequencies in healthy bearings, the most obvious being 33 k Hz, 22 k Hz, 10 k Hz, 5 k Hz, 1.9 k Hz. The higher the speed of the faulty bearing, the greater the vibration. The ball fault bearing also has four speed modes: increase, decrease, increase and decrease, and decrease and increase. There are three measurements for each mode, and the rotation speed is different during each measurement. There are 12 test data in total; these data are all converted into images of Hilbert’s marginal spectrum.

**Figure 11:**
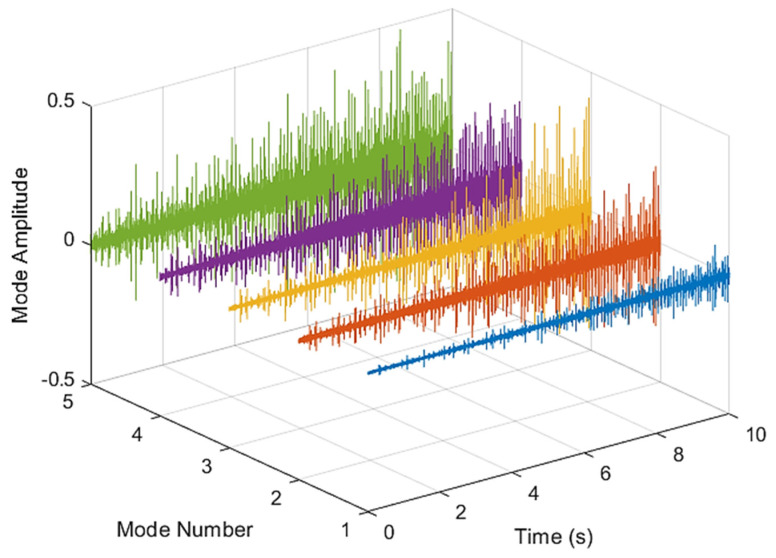
VMD analysis from a combined fault bearing under increasing rotational speed condition in time domain.

**Figure 12:**
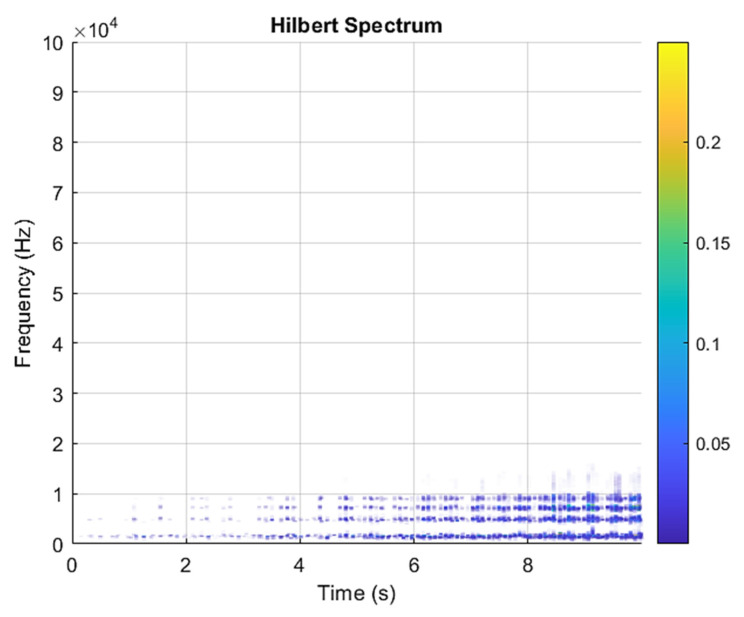
VMD analysis from a combined fault bearing under increasing rotational speed condition in Hilbert spectrum.

Figure 11 and Figure 12 are the VMD analysis results of the combined fault-bearing state, and Figure 11 is the time-domain waveform of the vibration signal’s VMD. Figure 12 is a component spectrum diagram; each state vibration signal is decomposed into four mode components. The results show that the IMF Hilbert marginal spectrum of the vibration data processed by VMD has a higher frequency resolution. There are four frequencies in healthy bearings, the most obvious being 9 k Hz, 7 k Hz, 5 k Hz, 1.5 k Hz. The higher the speed, the greater the vibration of the faulty bearing. The combined fault bearing has four speed modes: increase, decrease, increase and decrease, and decrease and increase. There are three measurements for each mode, and the rotation speed is different during each measurement. There are 12 test data in total. These data are all converted into images of Hilbert’s marginal spectrum.

In this study, all five categories of data, which included healthy, inner race fault, outer race fault, ball fault, and combined fault, were analyzed by VMD and converted into the Hilbert spectrum. Each category contained 12 test data; a total of 60 test data and 60 Hilbert spectrograms of VMD were obtained.

VMD time-domain waveform diagrams and component spectrograms of faults in different parts of the bearing are also different. This part is mainly to carry out the VMD of the fault vibration signals of different parts of the rolling bearing. In this way, the feature extraction of different parts of the bearing is realized, and finally, the diagnosis of the bearing fault is realized by comparing and analyzing the characteristic information of the healthy state and the fault state of different parts. From the analysis of the time-domain waveform diagram, it can be found that in the four failure states of the bearing, the vibration signal has a certain impact, and the frequency of each mode component is also different. It can be seen from the spectrogram of the four state components of the bearing that the vibration signal is processed by the VMD method. The bearing signals of different parts are effectively decomposed according to a certain bandwidth, and there is almost no mode aliasing between the mode components. By comparing and analyzing the component spectrograms under the four failure states, it is possible to simply analyze several failure states of the rolling bearing from the frequency distribution range, the energy level of the corresponding component spectrum, and the vibration intensity.

The IMF components obtained by VMD decomposition of the above healthy and four types of motor faults are subjected to Hilbert transformation, although the obtained Hilbert marginal spectra are different. However, because there are four different speed modes and the frequencies are close, engineers without professional training cannot understand the fault situation at first glance. In order to evaluate the method proposed in the text more comprehensively, it is compared with the current mainstream methods on the same test set, from both qualitative and quantitative aspects.

Efforts are also being made in the field of deep learning to promote the development of the miniaturization of neural networks. While ensuring the accuracy of the model, it is smaller and faster. This study has proposed a comparison of lightweight network models that make it possible for mobile terminals and embedded devices to run neural network models.

This research uses three deep learning image classification models/methods for identification: MobileNet, ShuffleNet, and DenseNet, to find the method with the highest recognition rate. Each category has only 12 images; in order to retain more images for verification testing, 60% of each category of images are trained, and 40% are verified. Therefore, there are seven images of each category for training and five images for verification testing. The size of the image in the training process is 224 × 224 × 3, and the pixels of the image will affect the training accuracy. The higher the pixel of the image, the higher the accuracy can be obtained, but the calculation time will increase.

**Figure 13:**
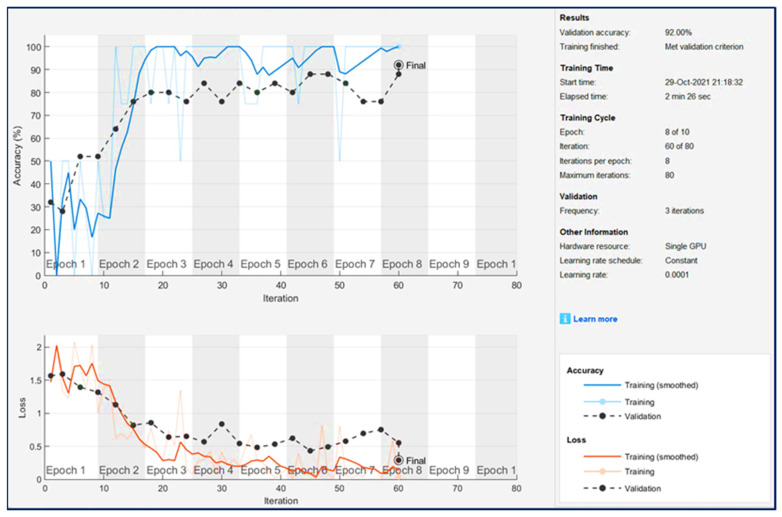
DenseNet training and verification network monitoring results.

By plotting various indicators during training, researchers can understand the training progress. For example, the figure can determine whether the accuracy of the network has improved and the speed at which it has improved, as well as whether the network has begun to overfit the training data. Figure 13 shows the results of DenseNet training and verification network monitoring. The figure demonstrates the following:

- Training accuracy—the classification accuracy of each mini-batch.
- Smooth training accuracy—Smooth training accuracy is obtained by applying a smoothing algorithm to training accuracy. It is less noisy than unsmoothed precision, and it is easier to spot trends.
- Validation accuracy—The classification accuracy of the entire validation set.
- Training loss, smooth training loss, and validation loss—the loss of each mini-batch, its smoothed version, and the loss of the validation set, respectively. If the last layer of the network is the classification layer, for example, then the loss function is the cross-entropy loss.

Once the training is complete, results are checked, which shows the final verification accuracy and reason for the end of the training. The final verification index is marked as Final in the drawing. After the training is over, the results are checked, which shows the final verification accuracy and the reason for the end of the training. The final verification index is marked as Final in the drawing. The figure on the right shows information about training time and settings.

In order to test the three methods with the same parameter settings, the specified algorithm, the Stochastic Gradient Descent (SGDM) optimizer with momentum, is used. The parameters can be explained as follows.

- Verbose is 0. Verbose is an indicator that displays training progress information. Verbose consists of 1 (true) and 0 (false).
- Verbose Frequency is 50. Frequency of verbose printing, which is the number of iterations between printing to the command window.
- Max epochs is 10. Max epochs is the maximum number of epochs. It is used for the maximum number of epochs of training. Iteration is a step in the gradient descent algorithm that uses small batch processing to minimize the loss function. An epoch is a full traversal of the training algorithm on the entire training set.
- Mini batch size is 4. Mini-batch size, the size of the mini-batch used for each training iteration. Mini-batch processing is a subset of the training set used to evaluate the gradient of the loss function and update the weights.
- Validation frequency is 3. Validation frequency is the frequency of network validation.
- Validation patience is 5. Validation patience is the patience of validation stopping.
- Initial learn rate is 0.0001. The initial learning rate is 0.01, but if the network training does not converge, you may wish to choose a smaller value. Learn rate schedule is none. Learn rate schedule is an option for dropping the learning rate during training.
- Learn rate drop period is 10. Learn rate drop period is number of epochs for dropping the learning rate.
- Learn rate drop factor is 0.1. Learn rate drop factor is the factor for dropping the learning rate.
- L2 Regularization is 0.0001. L2 Regularization is a factor for L2 regularization.
- Momentum is 0.9. Momentum is the contribution of the previous step.
- Gradient threshold is Inf. The gradient threshold can be Inf or a positive value. Gradient threshold method is L2 norm.
- Sequence length is longest. Sequence length fills the sequence in each mini-batch to make it the same length as the longest sequence. This option will not discard any data, but padding may cause noise to the network.
- Sequence padding value is 0. Sequence padding value is the value to pad input sequences.
- Execution environment is GPU. GPU is the hardware resource for training the network. Due to the popularity of deep learning, convolutional neural network models in the field of computer vision, such as MobileNet, are emerging in an endless stream, and the application of deep learning network models in image processing is improving. Neural networks are expanding, their structures are becoming more complex, and the hardware resources required for prediction and training are gradually increasing. Often, deep learning neural network models can only be run on servers with high computing power, and on mobile devices are difficult to run complex deep learning network models due to the limitations of hardware resources and computing power.

**Figure 14:**
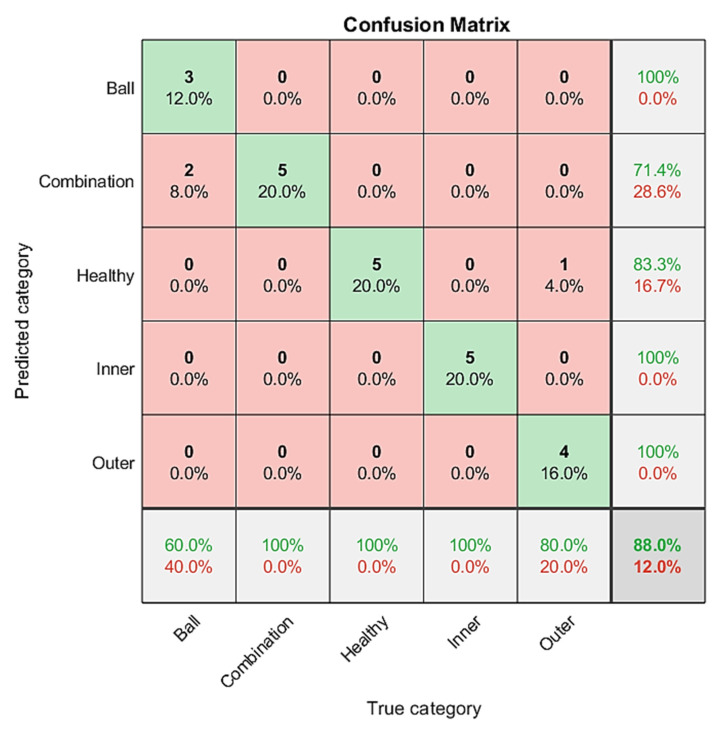
MobileNet classification results in confusion matrix; the accuracy rate is 88%.

The classification results of MobileNet are shown in Figure 14. The classification accuracy rate of the predicted five categories is as follows: 100% for ball fault bearing, inner race fault bearing, and outer race fault bearing; 71.4% for combination fault bearing; 83% for healthy bearing; and the total classification accuracy rate of 88%.

**Figure 15:**
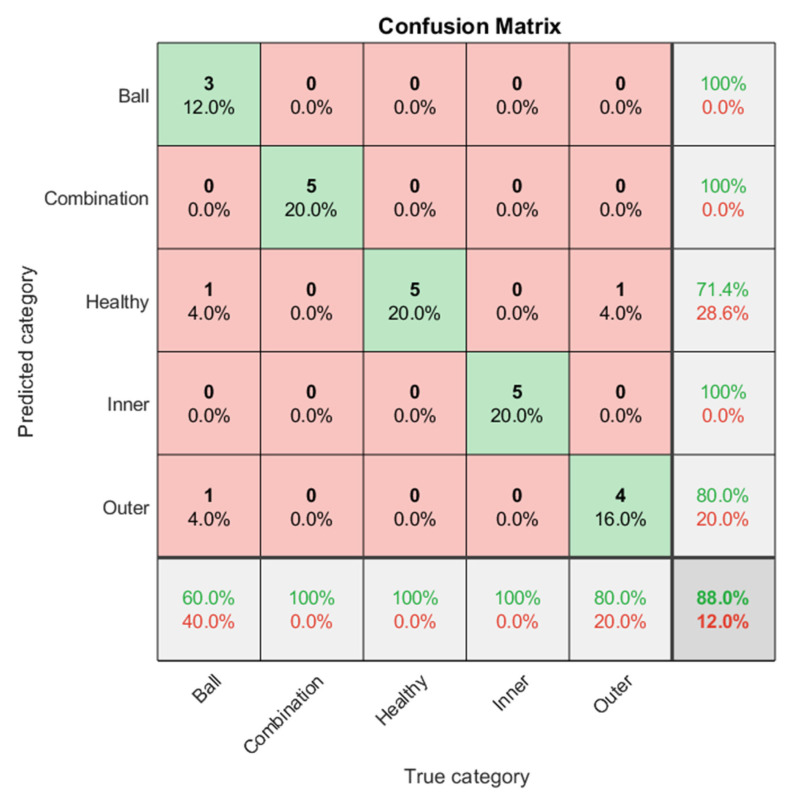
ShuffleNet classification results in confusion matrix; the accuracy rate is 88%.

The authors’ proposal is to use the ShuffleNet network and Point group convolution to improve the computational efficiency of convolution. The proposed channel shuffle operation can realize information exchange between different channels, which helps to encode more information. Compared with many other advanced network models, ShuffleNet greatly reduces the calculation cost and achieves excellent performance while ensuring calculation accuracy. In fact, grouped convolution was used in the AlexNet network model at the earliest, and some efficient neural network models such as Xception and MobileNet proposed later introduced deep separable convolution on the basis of grouped convolution. Although the ability of the model and the amount of calculation can be coordinated, the calculation amount of point-by-point convolution in the model occupies a large part. Therefore, the pixel-level group convolution is introduced in the ShuffleNet structure to reduce the computational complexity caused by the convolution operation. The ShuffleNet classification results are shown in Figure 15. The classification accuracy rate in the predicted three categories shows a classification accuracy rate of 100% for ball fault bearing, combination fault bearing, and inner race fault bearing; 71.4% for healthy bearing; 80% for outer race fault bearing; and a total classification accuracy rate of 88%.

Huang [31] proposed the use of DenseNet network following ResNet [32], which inherited the idea of residual network and improved the connection method. The DenseNet network takes image features as the starting point, and achieves better results, and reduces a large number of parameters through the reuse of image features. Instead of learning redundant features multiple times, feature reuse is a better feature extraction method. The advantages of DenseNet network compared to other deep networks are as follows:

(1) Compared with other deep network structures, it has fewer parameters.

(2) Based on the idea of residual network, the idea of feature reuse is added to the bypass.

(3) For network training, it prevents over-fitting, is easy to train, and has a certain regularization effect.

(4) The problem of vanishing gradient is alleviated.

There are many Dense block modules in the DenseNet network structure. In the Dense block module, the feature maps of different layers need to be connected. Therefore, the size of the feature maps in the Dense block must be kept the same.

**Figure 16:**
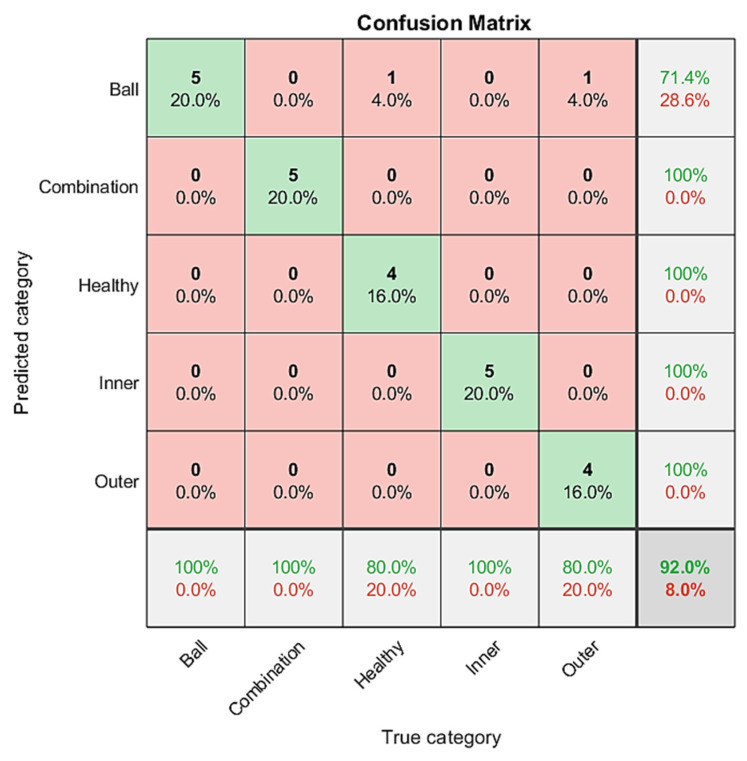
DenseNet classification results in confusion matrix; the accuracy rate is 92%.

The DenseNet classification results are shown in Figure 16. The classification accuracy rate of the predicted 100% in the five categories is 71.4% for combination fault bearing, healthy bearing, inner race fault bearing, outer race fault bearing, and ball fault bearing. The total classification accuracy rate is 92%.

In order to verify the computing time, this study compares the typical networks Alexnet, GooleNet, and ResNet with the three models of this study under the same standard, and shows the results in Table 2. The table compares the computing time and accuracy of the six models. All models have good classification prediction performance, but the DenseNet computing time is 146 s and the accuracy of 92% is the best in this study.

Based on the above research results, these three predictive classification methods have excellent performance, but the accuracy rate of DenseNet can reach 92%, which is the highest. The advantages of DenseNet are compared with other convolutional neural networks. DenseNet has excellent performance, mainly in the number of parameters, less calculation, and strong anti-fitting ability. DenseNet also has a strong anti-overfitting ability, which is suitable for network training when data is relatively scarce. Because the information flow and gradient flow in the entire network are improved, it is easy to train; the directly connected dense block structure itself has a regularization effect. It allows each layer to receive in-depth supervision and obtain gradient information from the loss function and input signal, which is more helpful for training deep network structures and is suitable for bearing fault diagnosis.

This study has some limitations. First, there must be sufficient data length. In this study, a total of 2,000,000 points were recorded for 10 s. If the data length is too small, VMD cannot present a complete Hilbert spectrogram, and deep learning cannot correctly classify it. Second, obtaining data must not be interfered with by noise. When the original signal is submerged in noise, it cannot be analyzed or is analyzed incorrectly. Third, the original limitation of VMD still exists, and will have mode mixing and end effect. Finally, the disadvantage of DenseNet is that training takes up a substantial amount of memory. Each splicing operation will open up a new memory to store the spliced features. This results in an *n*-layer network, which consumes the memory equivalent to *n*(*n* + 1)/2-layer network (the output of the *i*-th layer is stored in memory (*n* − *i* + 1)).

## 5. Conclusions

Modern industrial production equipment has made great contributions to improving productivity, saving natural and human resources, reducing the scrap rate, and ensuring product quality. Rotating machinery and equipment are developing in the direction of large volume, compact and complex structure, automation, and continuity of operation. Once it breaks down, if it cannot be shut down for maintenance in time, it will cause immeasurable economic losses to individuals and enterprises as well as unfavorable social repercussions. Equipment status monitoring, status early warning, and fault diagnosis technology can prevent, to a certain extent, such issues. This paper combines the requirements of bearing fault diagnosis and the characteristics of monitoring signals and attempts to introduce the existing deep neural network recognition model into bearing fault diagnosis. The research uses VMD to analyze the Hilbert spectrum conversion method to convert the one-dimensional bearing signal into a two-dimensional time-frequency diagram and combines it with the deep neural network DenseNet to realize intelligent fault diagnosis. VMD analyzes shows that the Hilbert spectrum contains the time-frequency domain contour characteristics of the fault signal and the fault location feature and combines the deep neural network to diagnose the fault. It uses a combination of time-frequency graphs and deep neural networks to realize high-accuracy and intelligent identification of faults. This research uses MobileNet, ShuffleNet, and DenseNet deep learning lightweight network classification prediction results. The data verification is divided into training set and test set samples, and a fault diagnosis model based on the VMD-DenseNet method is established. Finally, the deep neural network built by training and testing is used to obtain the diagnosis accuracy. DenseNet’s classification prediction accuracy rate was found to be 92%, ShuffleNet’s accuracy rate was 88%, and MobileNet’s accuracy rate was 88%. The proposed method does not require a large amount of prior knowledge of bearing fault diagnosis, including needing to denoise the signal, and simplifies the feature extraction process of bearing fault diagnosis, as well as has a high fault diagnosis accuracy rate. Recommendations for future research include the following. (1). Study the optimization of the parameters of the VMD and DenseNet algorithms; the use cases have different parameters and thus make for another topic. (2). This research database is verified by the University of Ottawa database, and more typical motor faults can be added for verification in future research. (3). In the future, there will be better time-frequency analysis and deep learning image classification algorithms, for which comparisons can be acquired to add to the evidence. (4). Some traditional methods, which also have advantages compared with the experimental results of the method in this study, can be further explored. (5). A more extensive statistical analysis of the experimental results could be performed to calculate and compare the confidence interval for the accuracy of the report.

## Figures and Tables

**Table 1:** Bearing health or failure and test conditions.

Bearing Health Conditions	Increasing Speed	Decreasing Speed	Increasing Then Decreasing Speed	Decreasing Then Increasing Speed
Healthy	H-A-1	H-B-1	H-C-1	H-D-1
	H-A-2	H-B-2	H-C-2	H-D-2
	H-A-3	H-B-3	H-C-3	H-D-3
Faulty (inner race fault)	I-A-1	I-B-1	I-C-1	I-D-1
	I-A-2	I-B-2	I-C-2	I-D-2
	I-A-3	I-B-3	I-C-3	I-D-3
Faulty (outer race fault)	O-A-1	O-B-1	O-C-1	O-D-1
	O-A-2	O-B-2	O-C-2	O-D-2
	O-A-3	O-B-3	O-C-3	O-D-3
Faulty (ball fault)	B-A-1	B-B-1	B-C-1	B-D-1
	B-A-2	B-B-2	B-C-2	B-D-2
	B-A-3	B-B-3	B-C-3	B-D-3
Faulty (combined fault)	C-A-1	C-B-1	C-C-1	C-D-1
	C-A-2	C-B-2	C-C-2	C-D-2
	C-A-3	C-B-3	C-C-3	C-D-3

**Table 2:** Comparing the computing time and accuracy of different models.

	Alexnet	GooleNet	ResNet	MobileNet	ShuffleNet	DenseNet
Computing time (s)	162	173	160	151	148	146
Accuracy (%)	80	84	84	88	88	92

## Data Availability

Not applicable.

## References

[1] Cempel C. (1988). Vibroacoustical diagnostics of machinery: An outline. Mech. Syst. Signal Process..

[2] Sturm A., Dipl-lng D.K. (1984). Diagnostics of rolling-element bearing condition by means of vibration monitoring under operating conditions. Measurement.

[3] Martin H.R., Honarvar F. (1995). Application of statistical moments to bearing failure detection. Appl. Acoust..

[4] Panda L.N., Panda P.K., Patro B.S. (1998). Diagnostics of antifriction bearings through statistical moments. Inverse Problems in Engineering Mechanics.

[5] Mechefske C.K., Mathew J. (1992). Fault detection and diagnosis in low speed rolling element bearings Part I: The use of parametric spectra. Mech. Syst. Signal Process..

[6] Logan D., Mathew J. (1996). Using the correlation dimension for vibration fault diagnosis of rolling element bearings—I. Basic concepts. Mech. Syst. Signal Process..

[7] Vapnik V., Guyon I., Hastie T. (1995). Support vector machines. Mach. Learn..

[8] Li Y., Xu M., Wei Y., Huang W. (2016). A new rolling bearing fault diagnosis method based on multiscale permutation entropy and improved support vector machine based binary tree. Measurement.

[9] Lin S.-L. (2021). Application of Machine Learning to a Medium Gaussian Support Vector Machine in the Diagnosis of Motor Bearing Faults. Electronics.

[10] Forrester B.D., Pussy H.C., Pussy S.C. (1990). Analysis of gear vibration in the time-frequency domain. Current Practices and Trends in Mechanical Failure Prevention.

[11] Huang N.E., Shen Z., Long S.R., Wu M.C., Shih H.H., Zheng Q., Yen N.-C., Tung C.-C., Liu H.H. (1998). The empirical mode decomposition and the Hilbert spectrum for nonlinear and non-stationary time series analysis. Proc. R. Soc. Lond. Ser. A Math. Phys. Eng. Sci..

[12] Wu Z., Huang N.E. (2004). A study of the characteristics of white noise using the empirical mode decomposition method. Proc. R. Soc. Lond. Ser. A Math. Phys. Eng. Sci..

[13] Han H., Cho S., Kwon S., Cho S.-B. (2018). Fault Diagnosis Using Improved Complete Ensemble Empirical Mode Decomposition with Adaptive Noise and Power-Based Intrinsic Mode Function Selection Algorithm. Electronics.

[14] Lee C.-Y., Hung C.-H. (2021). Feature Ranking and Differential Evolution for Feature Selection in Brushless DC Motor Fault Diagnosis. Symmetry.

[15] Wang L.H., Zhao X.P., Wu J.X., Xie Y.Y., Zhang Y.H. (2017). Motor fault diagnosis based on short-time Fourier transform and convolutional neural network. Chin. J. Mech. Eng..

[16] Li K., Ping X., Wang H., Chen P., Cao Y. (2013). Sequential Fuzzy Diagnosis Method for Motor Roller Bearing in Variable Operating Conditions Based on Vibration Analysis. Sensors.

[17] Gu K., Zhang Y., Liu X., Li H., Ren M. (2021). DWT-LSTM-Based Fault Diagnosis of Rolling Bearings with Multi-Sensors. Electronics.

[18] Nguyen C.D., Ahmad Z., Kim J.-M. (2021). Gearbox Fault Identification Framework Based on Novel Localized Adaptive Denoising Technique, Wavelet-Based Vibration Imaging, and Deep Convolutional Neural Network. Appl. Sci..

[19] Dragomiretskiy K., Zosso D. (2013). Variational mode decomposition. IEEE Trans. Signal Process..

[20] Liu Z., Chen X., He Z., Shen Z. (2013). LMD Method and Multi-Class RWSVM of Fault Diagnosis for Rotating Machinery Using Condition Monitoring Information. Sensors.

[21] Lin S.-L. (2021). Application Combining VMD and ResNet101 in Intelligent Diagnosis of Motor Faults. Sensors.

[22] Li X., Zhou K., Xue F., Chen Z., Ge Z., Chen X., Song K. (2020). A Wavelet Transform-Assisted Convolutional Neural Network Multi-Model Framework for Monitoring Large-Scale Fluorochemical Engineering Processes. Processes.

[23] Lee C.-Y., Cheng Y.-H. (2020). Motor Fault Detection Using Wavelet Transform and Improved PSO-BP Neural Network. Processes.

[24] Zamudio-Ramirez I., Osornio-Rios R.A., Antonino-Daviu J.A., Cureño-Osornio J., Saucedo-Dorantes J.-J. (2021). Gradual Wear Diagnosis of Outer-Race Rolling Bearing Faults through Artificial Intelligence Methods and Stray Flux Signals. Electronics.

[25] Chen C.-C., Liu Z., Yang G., Wu C.-C., Ye Q. (2021). An Improved Fault Diagnosis Using 1D-Convolutional Neural Network Model. Electronics.

[26] Ewert P., Kowalski C.T., Orlowska-Kowalska T. (2020). Low-Cost Monitoring and Diagnosis System for Rolling Bearing Faults of the Induction Motor Based on Neural Network Approach. Electronics.

[27] Skowron M., Orłowska-Kowalska T. (2020). Efficiency of Cascaded Neural Networks in Detecting Initial Damage to Induction Motor Electric Windings. Electronics.

[28] Chui K.T., Gupta B.B., Vasant P. (2021). A Genetic Algorithm Optimized RNN-LSTM Model for Remaining Useful Life Prediction of Turbofan Engine. Electronics.

[29] Howard A.G., Zhu M., Chen B., Kalenichenko D., Wang W., Weyand T., Andreetto M., Adam H. (2017). Mobilenets: Efficient convolutional neural networks for mobile vision applications. arXiv.

[30] Zhang X., Zhou X., Lin M., Sun J. Shufflenet: An extremely efficient convolutional neural network for mobile devices. Proceedings of the IEEE Conference on Computer Vision and Pattern Recognition.

[31] Huang G., Liu Z., Van Der Maaten L., Weinberger K.Q. Densely connected convolutional networks. Proceedings of the IEEE Conference on Computer Vision and Pattern Recognition.

[32] He K., Zhang X., Ren S., Sun J. Deep residual learning for image recognition. Proceedings of the IEEE Conference on Computer Vision and Pattern Recognition.

[33] Srivastava R.K., Greff K., Schmidhuber J. (2015). Highway networks. arXiv.

[34] Huang G., Sun Y., Liu Z., Sedra D., Weinberger K.Q. (2016). Deep networks with stochastic depth. European Conference on Computer Vision.

[35] Larsson G., Maire M., Shakhnarovich G. (2016). Fractalnet: Ultra-deep neural networks without residuals. arXiv.

